# Smaller, Stronger, More Stable: Peptide Variants of a SARS-CoV-2 Neutralizing Miniprotein

**DOI:** 10.3390/ijms23116309

**Published:** 2022-06-04

**Authors:** Lucas Weißenborn, Elie Richel, Helena Hüseman, Julia Welzer, Silvan Beck, Simon Schäfer, Heinrich Sticht, Klaus Überla, Jutta Eichler

**Affiliations:** 1 Department of Chemistry and Pharmacy, Friedrich-Alexander-Universität Erlangen-Nürnberg, 91058 Erlangen, Germany; lucas.weissenborn@fau.de (L.W.); helena.hh.hueseman@fau.de (H.H.); julia.welzer@fau.de (J.W.); silvan.beck@fau.de (S.B.); 2 Institute for Clinical and Molecular Virology, Universitätsklinikum Erlangen, Friedrich-Alexander-Universität Erlangen-Nürnberg, 91054 Erlangen, Germany; elie.richel@uk-erlangen.de (E.R.); klaus.ueberla@fau.de (K.Ü.); 3 Department of Biology, Genetics Division, Friedrich-Alexander-Universität Erlangen-Nürnberg, 91058 Erlangen, Germany; simon.schaefer@fau.de; 4 Institute of Biochemistry, Friedrich-Alexander-Universität Erlangen-Nürnberg, 91054 Erlangen, Germany; heinrich.sticht@fau.de

**Keywords:** SARS-CoV-2, virus, peptides, structure-based design, synthetic peptides, peptide mimics

## Abstract

Based on the structure of a de novo designed miniprotein (LCB1) in complex with the receptor binding domain (RBD) of the SARS-CoV-2 spike protein, we have generated and characterized truncated peptide variants of LCB1, which present only two of the three LCB1 helices, and which fully retained the virus neutralizing potency against different SARS-CoV-2 variants of concern (VOC). This antiviral activity was even 10-fold stronger for a cyclic variant of the two-helix peptides, as compared to the full-length peptide. Furthermore, the proteolytic stability of the cyclic peptide was substantially improved, rendering it a better potential candidate for SARS-CoV-2 therapy. In a more mechanistic approach, the peptides also served as tools to dissect the role of individual mutations in the RBD for the susceptibility of the resulting virus variants to neutralization by the peptides. As the peptides reported here were generated through chemical synthesis, rather than recombinant protein expression, they are amenable to further chemical modification, including the incorporation of a wide range of non-proteinogenic amino acids, with the aim to further stabilize the peptides against proteolytic degradation, as well as to improve the strength, as well the breadth, of their virus neutralizing capacity.

## 1. Introduction

As of May 2022, the SARS-CoV-2 pandemic has caused more than six million deaths worldwide (https://covid19.who.int/, accessed on 20 May 2022), clearly demonstrating the urgent need for efficient therapeutic and preventive measures to combat the disease and prevent infection with the virus, respectively. In the early phase of the pandemic, therapeutic options in the treatment of COVID-19 were largely limited to alleviating symptoms, mainly through anti-inflammatory intervention [1,2]. Complementary to vaccination, which is considered the most effective means of protecting against severe COVID-19, current therapeutic strategies against SARS-CoV-2 infection include a still low, but growing, number of drugs specifically targeting the virus. The first therapeutics approved for clinical use were monoclonal antibodies that recognize the spike protein, through which the virus contacts its receptor ACE2 expressed on lung epithelial cells [3,4]. Thus, binding of the antibodies to the spike protein prevents attachment of the virus to its host cell [5,6]. In addition, two orally available small molecules have recently been introduced as anti-SARS-CoV-2 drugs. Molnupiravir (Lagevrio^®^) increases the frequency of viral RNA mutations, impairing SARS-CoV-2 replication [7,8], while Nirmatrelvir (Paxlovid^®^) acts by inhibiting the SARS-CoV-2 main protease [9,10]. These two drugs are now available for the treatment of SARS-CoV-2-infected individuals who do not require supplemental oxygen, and who are at increased risk of developing severe COVID-19.

Interfering with the attachment of a virus to its host cell clearly is an effective means of preventing virus entry and, consequently, replication, as exemplified by the approved HIV-1 peptidic fusion inhibitor Enfuvirtide (Fuzeon^®^) [11]. Similar to antivirals targeting other steps of the viral replication cycle, this therapeutic approach is most effective when administered in an early phase of the disease, when infected individuals start to develop symptoms and are less likely to be hospitalized. Therapeutic monoclonal antibodies, however, which currently are the only available type of drugs acting as SARS-CoV-2 entry inhibitors, are typically administered through intravenous infusion in a hospital setting, and are, therefore, less likely to be used in early stages of the disease. Furthermore, antibodies are very large molecules, which may hamper tissue penetration, as well as access to sterically shielded epitopes. Therefore, current research is also focused on developing alternative, smaller molecules capable of interfering with the interaction of the SARS-CoV-2 spike protein with cellular ACE2 [12,13,14,15]. Over the past two years, a range of studies have reported the computational design of smaller proteins or peptides, either based on the structure of the two N-terminal α-helices of ACE2, through which it contacts the receptor binding domain (RBD) of the SARS-CoV-2 spike protein [16,17,18,19], or through de novo design based on the RBD structure [19,20,21]. In a mechanism similar to antibodies, such peptides and miniproteins can be expected to bind to RBD, preventing its interaction with ACE2, thus blocking infection. The sequence of one of these miniproteins, termed LCB1, is 55 amino acids long and folds into three distinct α-helices [19]. LCB1 was produced recombinantly and shown to bind to RBD, as well as efficiently inhibit SARS-CoV-2 infection of cells [19,22,23]. Furthermore, the 3D structure of an LCB1–RBD complex (pdb code 7JZU) revealed that LCB1 contacts regions of RBD, i.e., the receptor binding motif, identical to ACE2. Based on these promising features of LCB1, we used it as a blueprint to generate even smaller molecules, i.e., shorter peptides that present only one or two of the three LCB1 helices, and to evaluate their antiviral activity. Such truncation can be expected to further improve pharmacokinetic parameters, as well as reduce the risk of immunogenicity. Furthermore, these peptides are accessible through chemical synthesis, enabling the incorporation of non-proteinogenic amino acids, as well as other chemical modifications, which can be expected to increase the proteolytic stability of the molecule, improving its utility as a drug candidate [24,25].

## 2. Results and Discussion

### 2.1. Sequence Truncation and Cyclization

**Figure 1:**
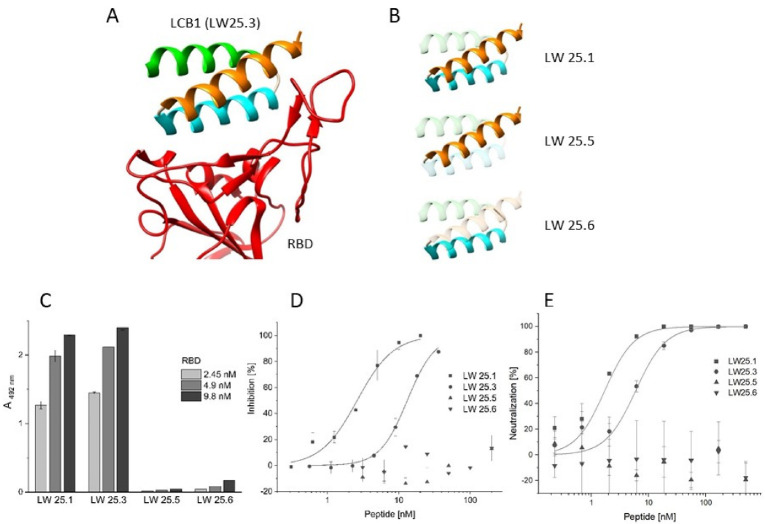
Design and functional characterization of peptides derived from LCB1. (**A**) Structure of the LCB1–RBD complex (pdb code 7JZU). (**B**) Depiction of LCB1 helices in different peptides. (**C**) Binding of RBD to peptides. (**D**) Inhibition of ACE2–RBD interaction by peptides. (**E**) Virus neutralization by peptides. See Materials and Methods for detail.

The 55 amino acid miniprotein LCB1 [19] had been designed through docking of large in silico miniprotein libraries against the RBD structure [26]. LCB1 folds into three consecutive α-helices, two of which interact with RBD, as evident from the cryo-electron microscopy structure of the LCB1–RBD complex (pdb code 7JZU). Addressing the question of whether all three helices of LCB1 are required for its interaction with RBD, we synthesized truncated variants that present helices 1 and 2 (LW25.1), as well as individual helix 1 (LW25.5) and helix 2 (LW25.6), respectively. These peptides, along with an N-terminally acetylated and C-terminally amidated variant of full-length LCB1 (LW25.3), were tested for their ability to inhibit the interaction of soluble recombinant ACE2 with RBD. Interestingly, the inhibitory activity of LW25.3 was essentially preserved in the two-helix peptide LW25.1 (Figure 1, Table 1), indicating that helix 3 is not required for its inhibitory activity. The individual helices (LW25.5 and LW25.6), on the other hand, were completely inactive, confirming the contribution of both helices to the interaction with RBD (Figure 1, Table 1). As a further step, C-terminal truncation of helix 2 in the two-helix peptide LW25.1 resulted in the identification of a 35-mer peptide (LW32.4), which fully retained the inhibitory activity of LW25.3 (Table 1). These results were confirmed in a neutralization assay using simian immunodeficiency virus (SIV) pseudotyped with the SARS-CoV-2 spike protein [27], in conjunction with A549 cells expressing human ACE2 and TMPRSS2. In this assay, both LW32.4 and LW25.1, as well as LW25.3, were shown to efficiently inhibit infection (Figure 1, Table 2), demonstrating the successful size reduction of LCB1 by approximately 30%.

**Figure 2:**
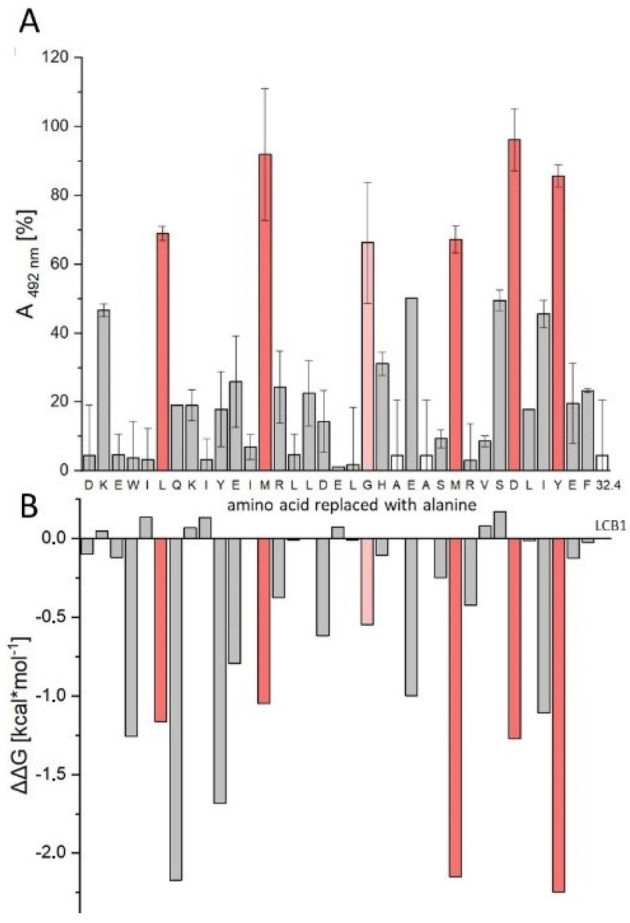
Experimental and computational alanine scan of LW32.4 and LCB1, respectively. (**A**) Inhibition of ACE2–RBD interaction by alanine exchange variants of LW32.4 (% remaining interaction at 12 nM peptide). Red bars indicate residues whose replacement with alanine resulted in more than 50% loss of inhibitory activity; white bars denote identical peptides. (**B**) Loss of binding free energy of the LCB1–RBD complex upon in silico replacement of individual LCB1 positions with alanine. See Materials and Methods for detail.

For the characterization of the interaction of the two-helix peptide LW32.4 with RBD at the level of individual amino acid residues, we generated an alanine scan, i.e., a set of peptide variants, in which all positions of the peptide were individually replaced with alanine. These peptides provided information on the contribution of each amino acid to the interaction with RBD. To compare the energy profiles of LW32.4 and LCB1, the experimental alanine scan data for LW32.4 were then matched with the results of an in silico alanine scan of LCB1. Testing the alanine scan peptides for inhibition of the ACE2–RBD interaction (Figure 2A) identified six residues, i.e., L6, M13, G20, M26, D30 and Y33, whose replacement with alanine resulted in more than 50% loss of inhibitory activity (red bars). All these positions are involved in the LCB1–RBD interface, according to the complex structure (pdb code 7JZU). Additional contact residues, however, including E3, Q7, Y10 and E23, can apparently be replaced with alanine without significant loss of affinity to RBD. Most of these residues (E3, Q7, E23) form specific hydrogen bonds with RBD in the complex structure 7JZU, suggesting that these interactions may be less stable in the shorter peptide LW32.4. The hot spot residues identified in the experimental alanine scan were also found in silico (Figure 2B), in addition to two more contact residues (Q7 and Y10), whose replacement with alanine was proposed to greatly destabilize the LCB1–RBD complex. In summary, the results obtained using the experimental alanine scan of the truncated peptide LW32.4, in comparison with the computational alanine scan of LCB1, strongly indicate a similar mechanism of interaction with RBD, which is the molecular basis of the inhibitory effect of these peptides on SARS-CoV-2 infection.

The 3D structure of the ACE2–LCB1 complex shows that the three LCB1 helices are interconnected by short loops composed of one (helix 1–2) and four (helix 2–3) amino acids, respectively, and aligned in an antiparallel fashion. Hence, the N-terminus of helix 1 is adjacent to the C-terminus of helix 2. This proximity is most pronounced between residues K2 and G39, which are only 8.7 Å apart. We, therefore, thought to stabilize this proximity covalently by replacing K2 and G39 in LW25.1 with cysteine and introducing a disulfide bridge between these two cysteine residues (peptide LW25.13). Interestingly, the inhibitory activity of this conformationally constrained peptide on the ACE2–RBD interaction was found to be approximately 20-fold and 9-fold, respectively, stronger than that of LW25.3 and LW32.4 (Table 1). A similar improvement was found for the virus neutralization capacity of LW25.13 (Table 2), indicating that introducing the covalent bridge between the N- and C-termini of the peptide had a stabilizing effect on its bioactive conformation.

### 2.2. Virus Variant Selectivity

In order to assess the breadth of the antiviral activity of LW25.3, LW32.4 and LW25.13, the peptides were tested in the ACE2–RBD inhibition and virus neutralization assays, respectively, using RBD proteins and pseudovirus from wild type (wt) virus, as well as the alpha, beta, delta and omicron variants of SARS-CoV-2. Interestingly, while the delta variant was susceptible to inhibition by LW25.3, LW32.4 and LW25.13, the beta and omicron variants were found to be largely resistant against all three peptides (Table 2). These two virus variants share specific mutations in the RBD, i.e., K417N/T, E484K/A and N501Y [28,29], suggesting that these positions are involved and critical for the interaction with LW25.3 and LW32.4, respectively. In fact, the structure of the LCB1–RBD complex indicates contacts of LCB1 with K417 and N501 of RBD. The only discrepancy in the inhibitory activity among the peptides was observed for the alpha virus variant, which, as previously reported for LCB1 [22,23], was well neutralized by LW25.3, but not by LW32.4. Re-introducing two positions (M36 and K37) at the C-terminus of the truncated peptide LW32.4, however, completely restored the inhibitory activity against the alpha variant (peptide LW32.2). Likewise, the cyclic peptide LW25.13, which also contains M36 and K37, was shown to neutralize the alpha variant at low nanomolar concentrations as well.

**Figure 3:**
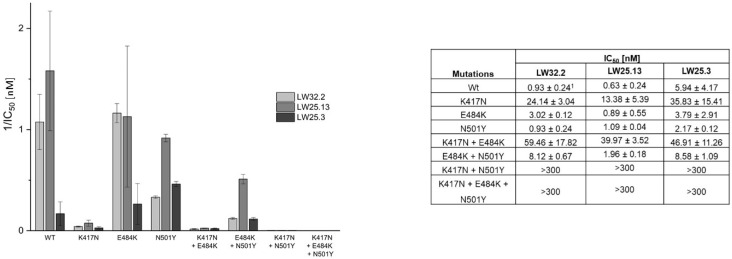
Impact of mutations in the RBD of SARS-CoV-2 spike protein on the susceptibility to virus neutralization by peptides. ^1^ Standard deviation based on at least three independent experiments.

For a detailed analysis of the relevance of the above-mentioned mutations in the RBD of various virus variants, these mutations were introduced either individually, or in combination, into the spike protein of wt SARS-CoV-2. The inhibitory effect of LW25.3, LW 32.2 and LW25.13 on pseudoviruses expressing these spike protein variants was then used to gauge the consequences of individual mutations, or combinations thereof (Figure 3). Interestingly, the effect of individual mutations E484K and N501N, respectively, on the neutralizing capacity of the peptides, appears to be negligible, as the IC_50_ values for these variants are not, or only slightly, different from those for wt virus. A combination of E484K and N501N resulted in an approximately three-fold (LW25.13), nine-fold (LW32.2) and 1.4-fold (LW25.3), respectively, reduction in inhibitory activity, which, however, was still in the low nanomolar range. A stronger effect was seen for the mutation K417N, which is present in the beta and omicron virus variants, and which decreased the susceptibility of the virus to inhibition by the peptides by a factor ranging from six (LW25.3) to 25 (LW32.2). The strong effect of the K417N exchange may be a consequence of the loss of a salt-bridge between K417 of RBD and D30 of LCB1-derived peptides. This effect was even more dramatic when K417N was combined with either E484K or N501N, or both, as virus variants containing these combinations of mutations could no longer efficiently be neutralized by any of the peptides. While the K417N + E484K variant was still neutralized, albeit with eight- to 64-fold higher IC_50_ values, the K417N + N501Y and K417N + E48K + N501Y variants were largely resistant to all three peptides, confirming the critical role of the K417N mutation, which, in combination with N501Y, abrogates susceptibility towards inhibition by the peptides. These two mutations are present in the beta and omicron variants of SARS-CoV-2, which may explain the resistance of these two variants against the peptides. Furthermore, the data illustrate the notion that it takes more than one mutation in the spike protein to confer a change in susceptibility of a virus variant for an inhibitor. Apart from that, it should also be noted that the cyclic peptide LW25.13 is consistently the most potent inhibitor among the tested peptides.

In conclusion, introducing a range of individual mutations, as well as combinations thereof, in the spike protein of SARS-CoV-2, in conjunction with our peptide inhibitors, we were able to dissect the contribution of these mutations to the loss of susceptibility to inhibition by the peptides, which helps elucidate the molecular basis of susceptibility of the various known SARS-CoV-2 variants to antiviral molecules.

### 2.3. Proteolytic Stability

**Figure 4:**
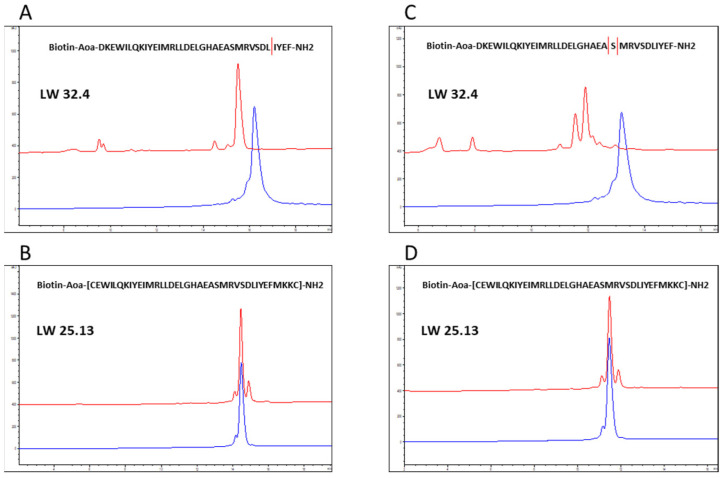
HPLC chromatograms of LW32.4 (**A**,**C**) and LW25.13 (**B**,**D**) before (blue curve) and after (red curve) 60 min. incubation with pepsin (**A**,**B**) and neutrophil elastase (**C**,**D**). See Section 3. Materials and Methods for experimental detail.

Oral availability of protein and peptide drugs is generally hampered by their inherent susceptibility to proteolytic degradation, as well as poor absorption due to their molecular size [30,31]. Therefore, these molecules are typically administered through intravenous, intramuscular or subcutaneous injection. Alternative parenteral administration routes include oral inhalation, which is preferred for the delivery of drugs to the lungs, as it results in high tissue levels shortly after application [31,32,33]. As SARS-CoV-2 primarily infects cells in the upper respiratory system and the lungs, an application of potential antiviral drugs for the treatment of SARS-CoV-2 infections through inhalation appears beneficial. While proteases are far less abundant in lung tissue, as compared to the gastro-intestinal tract, some proteases are also expressed by lung cells, including the primary cellular receptors of SARS-CoV-2, i.e., ACE2 and TMPRSS2, which are proteases presented on the surface of lung epithelial cells [34,35,36]. Furthermore, the aspartic endopeptidase cathepsin D is considered the main protease produced in lung tissue [36,37]. As the substrate specificity of cathepsin D is very similar to that of pepsin, which is far less expensive than cathepsin D, and is, therefore, often used as a cathepsin D surrogate in biomedical research [38,39,40,41], we examined the susceptibility of the linear peptide LW32.4 and the cyclic variant LW25.13 to proteolytic degradation by pepsin. Based on the preference of pepsin for peptide bonds with hydrophobic/aromatic amino acids at the P1 position, the sequence of the peptides contain a range of potential pepsin cleavage sites. Interestingly, though, LW32.4 was hydrolyzed only at position L31 (Figure 4A and Figure S2). More importantly, the cyclic peptide LW25.13 was shown to be largely stable towards pepsin for at least 60 min (Figure 4B), illustrating that cyclization of peptides can protect against degradation not only by exopeptidases, but also endopeptidases, such as pepsin.

Apparently, the more compact structure of the cyclic peptide LW25.13, as compared to LW32.4, shields the L31–I32 peptide bond in LW25.13, stabilizing it against attack by pepsin. This effect was also seen for neutrophil elastase, a protease expressed in inflamed lung tissue [36,42,43], which rapidly hydrolyzed LW32.4 (Figure 4C and Figure S3), while LW25.13 was still largely intact after 60 min exposure to the enzyme (Figure 4D). Based on these data, LW25.13 may be a better candidate, compared to the linear peptide LW32.4, for the development of a novel therapeutic approach against SARS-CoV-2 infection.

## 3. Materials and Methods

### 3.1. Peptide Synthesis

Peptides were synthesized as C-terminal amides by Fmoc/tBu-based solid-phase synthesis on TentaGel SRAM resin (110 mg, 0.23 mmol/g) using an automated multiple peptide synthesizer (ResPep SL by Intavis Inc.). In peptides containing disulfides, the N-terminal cysteine residue was coupled as Fmoc-Cys(StBu)-OH. In a standard coupling cycle, Fmoc-amino acid, HCTU and N-methyl morpholine (NMM) (all 5 eq.) in DMF were coupled twice for 7 min. Fmoc-His(Trt)-OH, Fmoc-Cys(Trt)-OH and Fmoc-Cys(StBu)-OH were coupled with DIC and ethyl cyanohydroxyiminoacetate (oxyma) (all 5 eq.) twice for 60 min. Each double coupling step was followed by a capping step with a mixture of acetic anhydride/pyridine/DMF (1:2:3; 2 min). The Fmoc groups were removed with 20% piperidine/DMF (5 min). The N-terminal amino groups were biotinylated (3 Eq. biotin, DIC and oxyma in N-Methylpyrrolidone). Peptides were cleaved from the resin by use of trifluoroacetic acid (TFA)/water/phenol/thioanisole/triisopropylsilane 80:5:5:5, precipitated in cold tert-butyl methyl ether, extracted with water and lyophilized. Peptides were purified by preparative HPLC (Phenomenex Kinetex C18 column, 100 × 21.2 mm, flow rate 30 mL/min, gradient of acetonitrile in H_2_O (both containing 0.1% TFA), 25–60% over 10 min). Disulfide bridges were formed after a first purification step, by disulfide exchange at 0.3 mg mL^−1^ in 50% acetonitrile in ammonium carbonate (pH 8, 0.1 M) for 12 h. The reaction was monitored by analytical HPLC with ESI mass spectrometry detection (LC-MS, conditions: Phenomenex Kinetex 2.6 μm C18 100 Å column, 50 × 2.1 mm, flow rate 0.55 mL/min, gradient of acetonitrile in H_2_O (both containing 0.1% TFA), 5–95% over 15 min.) Oxidized peptides were again purified by preparative HPLC. Stock solutions of purified peptides were prepared at 10 mM in DMSO. Concentrations were determined by UV spectroscopy (absorbance at 280 nm). For analytical data of purified peptides (LC/MS) see Supplementary Materials (Table S1, Figure S1).

### 3.2. Peptide–SARS-CoV-2 Spike Protein RBD Binding Assay

High binding Corning Costar microtiter half area plates were coated with 50 µL Neutravidin (ThermoFisher Scientific, Dreieich, Germany, 4.0 μg/mL) in phosphate buffer pH 7.2, overnight at 4 °C. After blocking with 110 µL 1% BSA in 0.1 M phosphate buffer, pH 7.2, for one hour, plates were incubated with 50 µL peptide solution (1 µM) for 15 min. Plates were then incubated for 3 h with 50 µL SARS-CoV-2 spike protein RBD (His-tagged, Sino Biological) in serial dilutions, starting at 39.2 nM. Bound RBD was detected with anti-His-HRP conjugate (Merck, Darmstadt, Germany, 0.025 µg/mL) for 30 min. Peptides and detection antibody were diluted in 0.1 M phosphate buffer, pH 7.2, containing 0.1% BSA and 0.01% Tween 20. Plates were washed four times with 0.01% Tween 20 in 0.1 M phosphate buffer, pH 7.2, after each incubation step. Plates were developed with 50 µL o-phenylenediamine (OPD) (1 mg/mL) in the presence of 0.03% H_2_O_2_ for 15 min in the dark. After the reaction was stopped with 25 µL 2 M H_2_SO_4_, absorbances were read at 492 nm.

### 3.3. SARS-CoV-2 Spike Protein RBD–hACE2 Inhibition Assay

High binding Corning Costar microtiter half area plates were coated with 50 µL of the respective SARS-CoV-2 spike protein RBD-Fc fusion protein (0.5 µg/mL, all RBD proteins from Sino Biological) in phosphate buffer pH 7.2, overnight at 4 °C. After blocking with 110 µL 1% BSA in 0.1 M phosphate buffer, pH 7.2, for one hour, plates were incubated with 50 µL peptide solution at serial dilutions, together with 50 µL His-tagged hACE2 (Sino Biological, Eschborn, Germany, 0.45 µg/mL) for three hours. Bound ACE2 was detected by incubation with anti-His-HRP conjugate (Sigma-Aldrich, 0.025 µg/mL) for 30 min. Peptides, hACE2 and detection antibody were diluted in 0.1 M phosphate buffer, pH 7.2, containing 0.1% BSA and 0.01% Tween 20. Plates were washed four times with 0.01% Tween 20 in 0.1 M phosphate buffer, pH 7.2, after each incubation step.

Plates were developed as described in 3.2. Percentage inhibition values were calculated using the following formula:

% Inhibition = (A_peptide_ − A_blank1_)/(A_reference_ − A_blank2_) × 100,

in which blank1 is a sample without spike RBD, reference is a sample without peptide, and blank2 is a sample with neither spike protein RBD, nor peptide. The program Origin Version 2020 (OriginLab Corporation) was used for curve fitting and calculation of IC_50_ values.

### 3.4. Virus Neutralization Assay/Generation of Virus Mutants

Generation of mutated SARS-CoV-2 Spikes: Point mutations were introduced into the wt spike expression plasmid by polymerase chain reaction (PCR). For that purpose, the wt spike DNA sequence was amplified by the Phusion^®^ High-Fidelity polymerase (New England Biolabs) using overlap extension of specific primers introducing the K417N, E484K or N501Y single mutations. After purification, the PCR fragments were digested with AgeI and Bsu36I (New England Biolabs) restriction enzymes and digested PCR products were gel purified and ligated into the wt spike expression plasmid using T4 DNA Ligase (ThermoFisher Scientific), according to the manufacturer recommendations. The multiple mutated SARS-CoV-2 spikes K417N + E484K, K417N + N501Y, E484K + N501Y and K417N + E484K + N501Y were generated by restriction enzymes digestion of the single mutated spike and ligated into the corresponding expression plasmids. XL-Gold^®^ Ultracompetent bacteria (Stratagene) were transformed with the ligation reactions and single colonies were screened for the respective mutations in the region encoding spike. All expected nucleotide substitutions in the wt spike DNA sequence were confirmed by sequencing.

Pseudoviruses neutralization assay: The neutralizing activity of the peptide variants was investigated by a lentiviral pseudotype assay, essentially as described previously [27,44]. In brief, pseudoviruses were produced by co-transfecting HEK293T cells with an expression plasmid encoding the respective spike variants, an SIV-based vector construct encoding luciferase and the SIV-based packaging plasmid. The supernatants of transfected cells containing the pseudoviruses were harvested, spun down for 5 min at 2000× *g* to pellet cell debris, 0.45 µM sterile filtered and stored at −20 °C for further use in neutralization assays. To investigate the peptide variants neutralization potency, 1.5 × 10^4^ A549 cells overexpressing human ACE2 and TMPRSS2 protease were seeded in 150 µL D10 medium into flat bottom 96-well plates one day before transduction. Serial dilutions of the peptide variants were performed in 60 µL D1.5 medium, followed by the addition of 60 µL of the respective lentiviral pseudovirus stock and incubated for 1 h at 37 °C. Subsequently, the previously seeded A549 cells were washed once with 200 µL phosphate buffer saline (PBS) per well, followed by the addition of 100 µL of the pseudoviruses: peptides mix and A549 cells were incubated for 48 h in the CO_2_ incubator at 37 °C. Finally, transduced A549 cells were washed twice with 200 µL PBS per well, and incubated with 50 µL ONE-Glo™ reagent (Promega) in 100 µL PBS for 3 min at RT. The luciferase intensity was, finally, measured on a microplate luminometer (VICTOR X5 from PerkinElmer, Rodgau, Germany). The luciferase activity from cells transduced with SIV pseudotyped particles lacking surface envelopes was subtracted as background from all sample values, while the luciferase intensity of cells transduced with pseudoviruses in absence of inhibitory molecules was considered as the reference value of transduction.

### 3.5. Analysis of Proteolytic Stability

The enzymes (pepsin, Ph.Eur. grade, Carl Roth GmbH, and neutrophil elastase, 20–22 U/mg, Abcam, respectively) were diluted to 5.5 µg/mL in TFA 10 mM (pepsin) or Tris buffer 0.1 M + NaCl 0.5 M pH 7.8 (neutrophil elastase) and pre-incubated for 15 min at 37 °C. Peptides were added at 100 µM and incubated at 37 °C. A sample of 40 µL was taken after 60 min of incubation and the reaction was stopped by adding 5 µL sodium carbonate solution (1 M, pepsin) and 5 µL TFA (neutrophil elastase), respectively. After 10 min, 60 µL acetonitrile/water 50:50 containing 1% TFA were added, and the resulting solution was analyzed by LC-MS (conditions: Phenomenex Kinetex 2.6 μm C18 100 Å column, 50 × 2.1 mm, flow rate 0.55 mL/min, gradient of acetonitrile in H_2_O (both containing 0.1% TFA, 20–60% over 20 min)).

### 3.6. In Silico Alanine Scan

The PDB structure of the LCB1–RBD complex (7JZU) was pre-processed utilizing the pdb-tools software kit [45] and the optimize tool of the FoldX forcefield [46]. The Positionscan Mutagenesis (PSSM) tool of FoldX [47] was used to mutate every residue to Ala and calculate the ΔΔG of interaction energy between protein and ligand upon mutation. Large negative ΔΔG differences correspond to interface residues, for which a replacement by alanine results in a significant destabilization of the protein–protein interface [48].

## 4. Conclusions

Based on a previously reported, computationally designed three-helix miniprotein (LCB1), we have generated and characterized truncated and conformationally constrained peptide variants of LCB1. The inhibitory effect on the interaction of the receptor binding domain (RBD) of the SARS-CoV-2 spike protein with ACE2, as well as the SARS-CoV-2 neutralizing potency, of the peptide presenting full length LCB1 (LW25.3) was shown to be maintained in LW25.1 and LW32.4, which present only two of the three helices of LCB1.

Moreover, the RBD–ACE2 inhibitory, as well as virus neutralizing capacity, of a cyclic variant (LW25.13) of the truncated peptides was found to be approximately ten-fold stronger than that of LW25.3. This cyclic peptide was also shown to be considerably more stable towards proteases, such as pepsin and neutrophil elastase, as compared to the linear peptide LW32.4. Taken together, these data illustrate how the activity, as well as stability, of bioactive peptides can be improved through chemical modification, such as truncation and cyclization.

Furthermore, using these peptides in conjunction with synthetic virus variants containing individual mutations, or combinations thereof, we were able to dissect the role of these mutations, which are present in various virus variants of concern, to the susceptibility of the virus to neutralization by the peptides.

Finally, as the peptides reported here were generated through chemical synthesis, rather than recombinant protein synthesis, they are amenable to further chemical modification, including the incorporation of a wide range of non-proteinogenic amino acids. Such modifications can be expected to further stabilize the peptides against proteolytic degradation, as well as to improve the strength, and the breadth, of their virus neutralizing capacity.

## Figures and Tables

**Table 1:** Peptide sequences and inhibitory effect on the ACE2–RBD interaction.

Peptide	Sequence	IC_50_ [nM]
LW25.3	Biotin-Aoa ^2^-DKEWILQKIYEIMRLLDELGHAEASMRVSDLIYEFMKKGDERLLEEAERLLEEVE-NH_2_	12.94 ± 0.19 ^1^
LW25.1	Biotin-Aoa-DKEWILQKIYEIMRLLDELGHAEASMRVSDLIYEFMKKG-NH_2_	2.62 ± 0.21
LW32.2	Biotin-Aoa-DKEWILQKIYEIMRLLDELGHAEASMRVSDLIYEFMK-NH_2_	1.66 ± 0.09
LW32.4	Biotin-Aoa-DKEWILQKIYEIMRLLDELGHAEASMRVSDLIYEF-NH_2_	6.02 ± 0.53
LW25.13	Biotin-Aoa-[^3^ CEWILQKIYEIMRLLDELGHAEASMRVSDLIYEFMKKC]-NH_2_	0.65 ± 0.23
LW25.5	Biotin-Aoa-DKEWILQKIYEIMRLLDEL-NH_2_	>200
LW25.6	Biotin-Aoa-GHAEASMRVSDLIYEFMKKG-NH_2_	>200

^1^ standard deviation based on at least three independent experiments; ^2^ Aoa, 8-amino-3,6-dioxa-octanoic acid; ^3^ square brackets indicate a disulfide bridge between cysteine residues.

**Table 2:** Virus variant selectivity of LCB1-derived peptides.

Peptide	Inhibition of ACE2–RBD Interaction; IC_50_ [nM]				
	**wt**	**Alpha**	**Beta**	**Delta**	**Omicron**
LW 25.3	12.94 ± 0.19 ^1^	2.48 ± 0.10	n.d. ^2^	1.40 ± 0.03	>100
LW25.1	2.62 ± 0.21	1.78 ± 0.15	n.d.	0.78 ± 0.01	>100
LW32.4	6.02 ± 0.53	>100	n.d.	6.21 ± 0.91	>100
LW32.2	1.66 ± 0.09	1.74 ± 0.11	n.d	0.77 ± 0.01	>100
LW25.13	0.65 ± 0.23	0.85 ± 0.05	n.d.	0.92 ± 0.01	>100
	**Virus neutralization; IC_50_ [nM]**				
LW 25.3	5.94 ± 4.17	13.31 ± 4.55	>500	4.52 ± 0.43	>500
LW25.1	1.70 ± 0.11	8.38 ± 3.31	>500	0.71 ± 0.23	>500
LW32.4	11.78 ± 3.50	>500	>500	10.17 ± 1.78	>500
LW32.2	0.93 ± 0.24	6.15 ± 5.96	>500	1.54 ± 0.23	>500
LW25.13	0.63 ± 0.24	1.35 ± 0.08	>500	0.68 ± 0.12	>500

^1^ standard deviation, based on at least three independent experiments; ^2^ n.d., not determined.

## Data Availability

Not applicable.

## Supplementary Materials

The following supporting information can be downloaded at: https://www.mdpi.com/article/10.3390/ijms23116309/s1.

## References

[1] Tobaiqy M., Qashqary M., Al-Dahery S., Mujallad A., Hershan A.A., Kamal M.A., Helmi N. (2020). Therapeutic management of patients with COVID-19: A systematic review. Infect. Prev. Pract..

[2] Soy M., Keser G., Atagündüz P., Tabak F., Atagündüz I., Kayhan S. (2020). Cytokine storm in COVID-19: Pathogenesis and overview of anti-inflammatory agents used in treatment. Clin. Rheumatol..

[3] Taylor P.C., Adams A.C., Hufford M.M., De La Torre I., Winthrop K., Gottlieb R.L. (2021). Neutralizing monoclonal antibodies for treatment of COVID-19. Nat. Rev. Immunol..

[4] Song W., Gui M., Wang X., Xiang Y. (2018). Cryo-EM structure of the SARS coronavirus spike glycoprotein in complex with its host cell receptor ACE2. PLoS Pathog..

[5] Tuccori M., Convertino I., Ferraro S., Valdiserra G., Cappello E., Fini E., Focosi D. (2021). An overview of the preclinical discovery and development of bamlanivimab for the treatment of novel coronavirus infection (COVID-19): Reasons for limited clinical use and lessons for the future. Expert Opin. Drug Discov..

[6] Peter A.S., Roth E., Schulz S.R., Fraedrich K., Steinmetz T., Damm D., Hauke M., Richel E., Mueller-Schmucker S., Habenicht K. (2021). A pair of noncompeting neutralizing human monoclonal antibodies protecting from disease in a SARS-CoV-2 infection model. Eur. J. Immunol..

[7] Malone B., Campbell E.A. (2021). Molnupiravir: Coding for catastrophe. Nat. Struct. Mol. Biol..

[8] Kabinger F., Stiller C., Schmitzová J., Dienemann C., Kokic G., Hillen H.S., Höbartner C., Cramer P. (2021). Mechanism of molnupiravir-induced SARS-CoV-2 mutagenesis. Nat. Struct. Mol. Biol..

[9] Halford B. (2022). The Path to Paxlovid. ACS Cent. Sci..

[10] Owen D.R., Allerton C.M.N., Anderson A.S., Aschenbrenner L., Avery M., Berritt S., Boras B., Cardin R.D., Carlo A., Coffman K.J. (2021). An oral SARS-CoV-2 M(pro) inhibitor clinical candidate for the treatment of COVID-19. Science.

[11] Matthews T., Salgo M., Greenberg M., Chung J., Demasi R., Bolognesi D. (2004). Enfuvirtide: The first therapy to inhibit the entry of HIV-1 into host CD4 lymphocytes. Nat. Rev. Drug Discov..

[12] Wang L., Wu Y., Yao S., Ge H., Zhu Y., Chen K., Chen W.-Z., Zhang Y., Zhu W., Wang H.-Y. (2021). Discovery of potential small molecular SARS-CoV-2 entry blockers targeting the spike protein. Acta Pharmacol. Sin..

[13] Sadremomtaz A., Al-Dahmani Z.M., Ruiz-Moreno A.J., Monti A., Wang C., Azad T., Bell J.C., Doti N., Velasco-Velázquez M.A., De Jong D. (2022). Synthetic Peptides That Antagonize the Angiotensin-Converting Enzyme-2 (ACE-2) Interaction with SARS-CoV-2 Receptor Binding Spike Protein. J. Med. Chem..

[14] Hakmi M., Bouricha E.L.M., Akachar J., Lmimouni B., El Harti J., Belyamani L., Ibrahimi A. (2022). In silico exploration of small-molecule α-helix mimetics as inhibitors of SARS-CoV-2 attachment to ACE2. J. Biomol. Struct. Dyn..

[15] Karoyan P., Vieillard V., Gómez-Morales L., Odile E., Guihot A., Luyt C.-E., Denis A., Grondin P., Lequin O. (2021). Human ACE2 peptide-mimics block SARS-CoV-2 pulmonary cells infection. Commun. Biol..

[16] Rajpoot S., Solanki K., Kumar A., Zhang K.Y.J., Pullamsetti S.S., Savai R., Faisal S.M., Pan Q., Baig M.S. (2022). In-Silico Design of a Novel Tridecapeptide Targeting Spike Protein of SARS-CoV-2 Variants of Concern. Int. J. Pept. Res. Ther..

[17] Choudhury A.R., Maity A., Chakraborty S., Chakrabarti R. (2022). Computational Design of Stapled Peptide Inhibitor against SARS-CoV-2 Receptor Binding Domain. Pep. Sci..

[18] Sitthiyotha T., Chunsrivirot S. (2021). Computational design of SARS-CoV-2 peptide binders with better predicted binding affinities than human ACE2 receptor. Sci. Rep..

[19] Cao L., Goreshnik I., Coventry B., Case J.B., Miller L., Kozodoy L., Chen R.E., Carter L., Walls A.C., Park Y.-J. (2020). De novo design of picomolar SARS-CoV-2 miniprotein inhibitors. Science.

[20] Huang X., Pearce R., Zhang Y. (2020). De novo design of protein peptides to block association of the SARS-CoV-2 spike protein with human ACE2. Aging.

[21] Cosic I., Kuhar U., Krapez U., Slavec B. (2022). De Novo Designed Peptide to Prevent SARS-CoV-2 Interaction with ACE2 Receptor on Host Cells. Int. J. Sci..

[22] Case J.B., Chen R.E., Cao L., Ying B., Winkler E.S., Goreshnik I., Shrihari S., Kafai N.M., Bailey A.L., Xie X. (2021). Ultrapotent miniproteins targeting the receptor-binding domain protect against SARS-CoV-2 infection and disease in mice. Cell Host Microbe.

[23] Hunt A.C., Case J.B., Park Y.J., Cao L., Wu K., Walls A.C., Liu Z., Bowen J.E., Yeh H.W., Saini S. (2022). Multivalent designed proteins neutralize SARS-CoV-2 variants of concern and confer protection against infection in mice. Sci. Transl. Med..

[24] Van Witteloostuijn S.B., Pedersen S.L., Jensen K.J. (2016). Half-Life Extension of Biopharmaceuticals using Chemical Methods: Alternatives to PEGylation. ChemMedChem.

[25] Kontermann R.E. (2016). Half-life extended biotherapeutics. Expert Opin. Biol. Ther..

[26] Lan J., Ge J., Yu J., Shan S., Zhou H., Fan S., Zhang Q., Shi X., Wang Q., Zhang L. (2020). Structure of the SARS-CoV-2 spike receptor-binding domain bound to the ACE2 receptor. Nature.

[27] Dispinseri S., Secchi M., Pirillo M.F., Tolazzi M., Borghi M., Brigatti C., De Angelis M.L., Baratella M., Bazzigaluppi E., Venturi G. (2021). Neutralizing antibody responses to SARS-CoV-2 in symptomatic COVID-19 is persistent and critical for survival. Nat. Commun..

[28] Sanches P.R.S., Charlie-Silva I., Braz H.L.B., Bittar C., Freitas Calmon M., Rahal P., Cilli E.M. (2021). Recent advances in SARS-CoV-2 Spike protein and RBD mutations comparison between new variants Alpha (B.1.1.7, United Kingdom), Beta (B.1.351, South Africa), Gamma (P.1, Brazil) and Delta (B.1.617.2, India). J. Virus Erad..

[29] Wang L., Cheng G. (2022). Sequence analysis of the emerging SARS-CoV-2 variant Omicron in South Africa. J. Med. Virol..

[30] Zhu Q., Chen Z., Paul P.K., Lu Y., Wu W., Qi J. (2021). Oral delivery of proteins and peptides: Challenges, status quo and future perspectives. Acta Pharm. Sin. B.

[31] Zelikin A.N., Ehrhardt C., Healy A.M. (2016). Materials and methods for delivery of biological drugs. Nat. Chem..

[32] Frohlich E., Salar-Behzadi S. (2021). Oral inhalation for delivery of proteins and peptides to the lungs. Eur. J. Pharm. Biopharm..

[33] Liang W., Pan H.W., Vllasaliu D., Lam J.K.W. (2020). Pulmonary Delivery of Biological Drugs. Pharmaceutics.

[34] Bosso M., Thanaraj T.A., Abu-Farha M., Alanbaei M., Abubaker J., Al-Mulla F. (2020). The Two Faces of ACE2: The Role of ACE2 Receptor and Its Polymorphisms in Hypertension and COVID-19. Mol. Ther.-Methods Clin. Dev..

[35] Laporte M., Naesens L. (2017). Airway proteases: An emerging drug target for influenza and other respiratory virus infections. Curr. Opin. Virol..

[36] Woods A., Andrian T., Sharp G., Bicer E.M., Vandera K.-K.A., Patel A., Mudway I., Dailey L.A., Forbes B. (2020). Development of new in vitro models of lung protease activity for investigating stability of inhaled biological therapies and drug delivery systems. Eur. J. Pharm. Biopharm..

[37] Benes P., Vetvicka V., Fusek M. (2008). Cathepsin D—Many functions of one aspartic protease. Crit. Rev. Oncol./Hematol..

[38] Ferguson J.B., Andrews J.R., Voynick I.M., Fruton J.S. (1973). The Specificity of Cathepsin D. J. Biol. Chem..

[39] Rawlings N.D., Barrett A.J., Thomas P.D., Huang X., Bateman A., Finn R.D. (2018). The MEROPS database of proteolytic enzymes, their substrates and inhibitors in 2017 and a comparison with peptidases in the PANTHER database. Nucleic Acids Res..

[40] Athaudaa S.B., Takahashia K. (2002). Cleavage specificities of aspartic proteinases toward oxidized insulin B chain at different pH values. Protein Pept. Lett..

[41] Bateman A., Martin M.-J., Orchard S., Magrane M., Agivetova R., Ahmad S., Alpi E., Bowler-Barnett E.H., Britto R., Bursteinas B. (2021). UniProt: The universal protein knowledgebase in 2021. Nucleic Acids Res..

[42] Moraes T.J., Chow C.W., Downey G.P. (2003). Proteases and lung injury. Crit. Care Med..

[43] Greene C.M., McElvaney N.G. (2009). Proteases and antiproteases in chronic neutrophilic lung disease—Relevance to drug discovery. Br. J. Pharmacol..

[44] Lapuente D., Fuchs J., Willar J., Vieira Antão A., Eberlein V., Uhlig N., Issmail L., Schmidt A., Oltmanns F., Peter A.S. (2021). Protective mucosal immunity against SARS-CoV-2 after heterologous systemic prime-mucosal boost immunization. Nat. Commun..

[45] Rodrigues J.P.G.L.M., Teixeira J.M.C., Trellet M., Bonvin A.M.J.J. (2018). pdb-tools: A swiss army knife for molecular structures. F1000Research.

[46] Caldararu O., Blundell T.L., Kepp K.P. (2021). A base measure of precision for protein stability predictors: Structural sensitivity. BMC Bioinform..

[47] Delgado J., Radusky L.G., Cianferoni D., Serrano L. (2019). FoldX 5.0: Working with RNA, small molecules and a new graphical interface. Bioinformatics.

[48] Moreira I.S., Fernandes P.A., Ramos M.J. (2007). Computational alanine scanning mutagenesis—An improved methodological approach. J. Comput. Chem..

