# Supplementary material for: Smaller, Stronger, More Stable: Peptide Variants of a SARS-CoV-2 Neutralizing Miniprotein
Source: Int J Mol Sci. 2022 Jun 4;23(11):6309. doi: 10.3390/ijms23116309 (PMC9181698; doi:10.3390/ijms23116309)
Supplement: Supplementary file 1 [file ijms-23-06309-s001.zip › ijms-1733367-supplementary.pdf]

<u>Supplementary Materials</u>

Smaller, stronger, more stable: Peptide variants of a SARS-CoV-2 neutralizing miniprotein

Lucas Weißenborn [1], Elie Richel [2], Helena Hüseman [1], Julia Welzer [1], Silvan Beck [1], Simon Schäfer [3], Heinrich Sticht [4], Klaus Überla [2] and Jutta Eichler [1],*

[1]Department of Chemistry and Pharmacy, Friedrich-Alexander-Universität Erlangen-Nürnberg, Germany

[2]Institute for Clinical and Molecular Virology; Universitätsklinikum Erlangen, Fried-rich-Alexander-Universität Erlangen-Nürnberg, Germany

[3]Department of Biology, Genetics Division, Friedrich-Alexander-Universität Erlangen-Nürnberg, Germany

[4]Institute of Biochemistry, Friedrich-Alexander-Universität Erlangen-Nürnberg, Germany

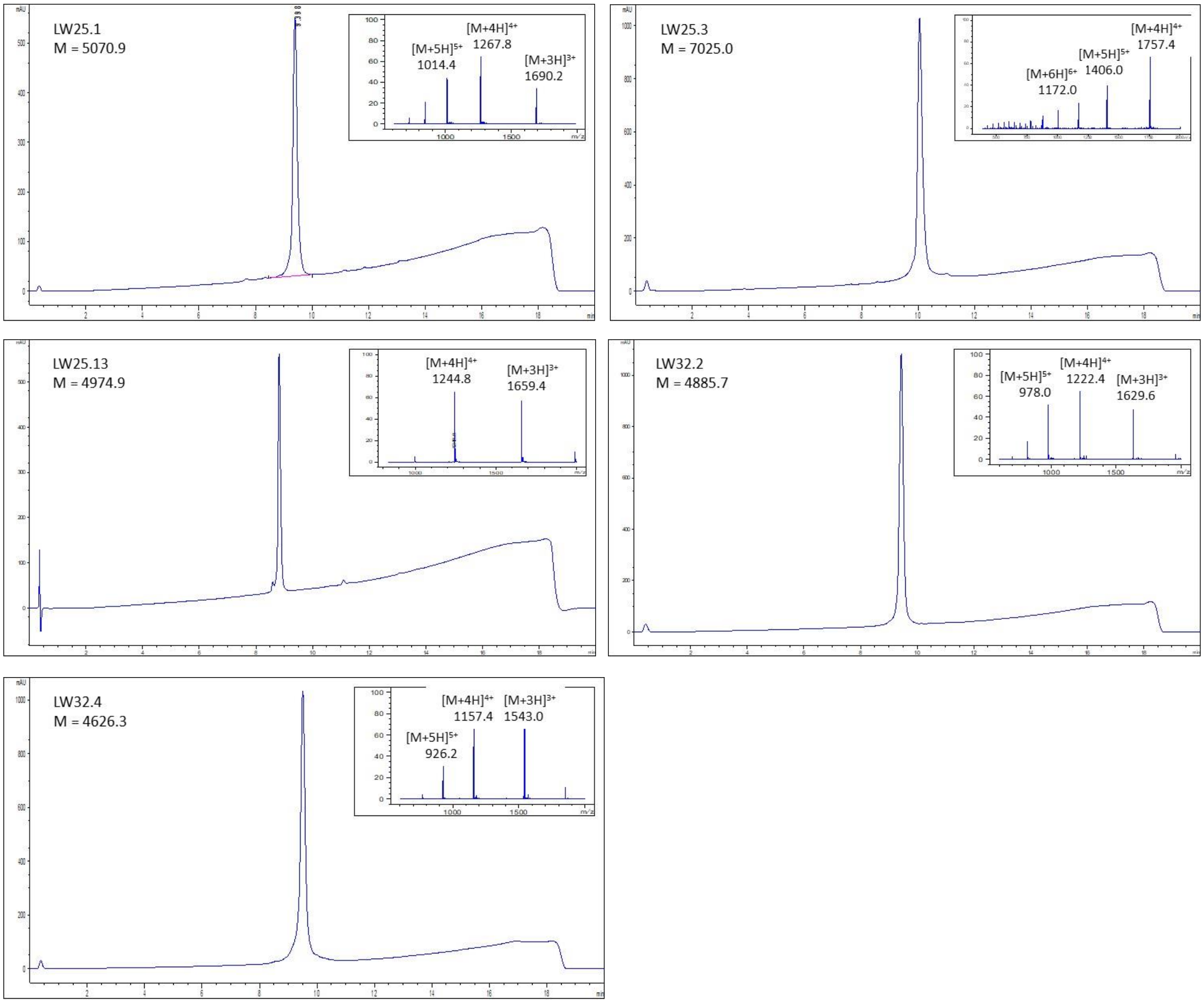

Figure S1: HPLC chromatograms and ESI-mass spectra (insets) of purified peptides.

Table S1. ESI-mass spectrometry data of LW32.4 variants.

D-Scan	Modification	MW calc.	$[M+5H]^{5+}$	$[M+4H]^{4+}$	$[M+3H]^{3+}$
LW33.1	D1d	4626.391	926.4	1157.6	1543.2
LW33.2	K2k	4626.391	926.2	1157.6	1543.0
LW33.3	E3e	4626.391	926.2	1157.6	1543.2
LW33.4	W4w	4626.391	926.2	1157.6	1543.0
LW33.5	I5i	4626.391	926.4	1157.8	1543.2
LW33.6	L6l	4626.391	926.2	1157.6	1543.0
LW33.7	Q7q	4626.391	926.2	1157.6	1543.0
LW33.8	K8k	4626.391	926.2	1157.6	1543.0
LW33.9	I9i	4626.391	926.2	1157.6	1543.0
LW33.10	Y10y	4626.391	926.2	1157.6	1543.0
LW33.11	E11e	4626.391	926.2	1157.6	1543.0
LW33.12	I12i	4626.391	926.2	1157.6	1543.0
LW33.13	M13m	4626.391	926.2	1157.6	1543.0
LW33.14	R14r	4626.391	926.2	1157.4	1543.0
LW33.15	L15l	4626.391	926.2	1157.6	1543.0
LW33.16	L16l	4626.391	926.2	1157.6	1543.0
LW33.17	D17d	4626.391	926.2	1157.6	1543.0
LW33.18	E18e	4626.391	926.2	1157.6	1543.0
LW33.19	L19l	4626.391	926.2	1157.6	1543.0
LW33.21	H21h	4626.391	926.2	1157.6	1543.2
LW33.22	A22a	4626.391	926.2	1157.6	1543.2
LW33.23	E23e	4626.391	926.2	1157.6	1543.0
LW33.24	A24a	4626.391	926.4	1157.8	1543.2
LW33.25	S25s	4626.391	926.2	1157.6	1543.2
LW33.26	M26m	4626.391	926.2	1157.6	1543.0
LW33.27	R27r	4626.391	926.2	1157.6	1543.0
LW33.28	V28v	4626.391	926.2	1157.6	1543.2
LW33.29	S29s	4626.391	926.4	1157.6	1543.0
LW33.30	D30d	4626.391	926.2	1157.6	1543.2
LW33.31	L31l	4626.391	926.2	1157.4	1543.0
LW33.32	I32i	4626.391	926.2	1157.6	1543.2
LW33.33	Y33y	4626.391	926.2	1157.6	1543.0
LW33.34	E34e	4626.391	926.2	1157.6	1543.0
LW33.35	F35f	4626.391	926.2	1157.4	1543.0

Ala-Scan	Modification	MW calc.	$[M+5H]^{5+}$	$[M+4H]^{4+}$	$[M+3H]^{3+}$
LW38.1	D1A	4582.382	917.4	1146.6	1528.4
LW38.2	K2A	4569.295	914.8	1143.2	1524.0
LW38.3	E3A	4568.355	914.6	1143.0	1523.8
LW38.4	W4A	4511.256	903.2	1128.8	1504.8

LW38.5	I5A	4584.310	917.8	1147.2	1529.0
LW38.6	L6A	4584.310	917.8	1147.0	1529.2
LW38.7	Q7A	4569.339	914.8	1143.4	1524.2
LW38.8	K8A	4569.295	914.8	1143.2	1524.0
LW38.9	I9A	4584.310	917.8	1147.2	1529.0
LW38.10	Y10A	4534.294	907.8	1134.6	1512.4
LW38.11	E11A	4568.355	914.6	1143.0	1523.8
LW38.12	I12A	4584.310	917.8	1147.0	1529.2
LW38.13	M13A	4566.277	914.2	1142.6	1523.0
LW38.14	R14A	4541.281	909.4	1136.4	1514.8
LW38.15	L15A	4584.310	917.8	1147.0	1529.2
LW38.16	L16A	4584.310	917.8	1147.0	1529.0
LW38.17	D17A	4582.382	917.4	1146.6	1528.2
LW38.18	E18A	4568.355	914.6	1143.0	1523.6
LW38.19	L19A	4584.310	917.8	1147.0	1528.8
LW38.20	G20A	4640.418	929.0	1164.0	1547.6
LW38.21	H21A	4560.328	913.2	1141.0	1521.2
LW38.22	E23A	4568.355	914.6	1143.0	1523.6
LW38.23	S25A	4610.392	923.0	1153.6	1537.6
LW38.24	M26A	4566.277	914.2	1142.6	1522.8
LW38.25	R27A	4541.281	909.2	1136.2	1514.4
LW38.26	V28A	4598.377	920.6	1150.6	1533.6
LW38.27	S29A	4610.392	923.0	1153.4	1537.6
LW38.28	D30A	4582.382	917.4	1146.6	1528.2
LW38.29	I32A	4584.310	917.8	1147.0	1528.8
LW38.30	Y33A	4534.294	907.8	1134.6	1512.2
LW38.31	E34A	4568.355	914.6	1143.0	1523.6
LW38.32	F35A	4550.293	911.0	1138.4	1517.6

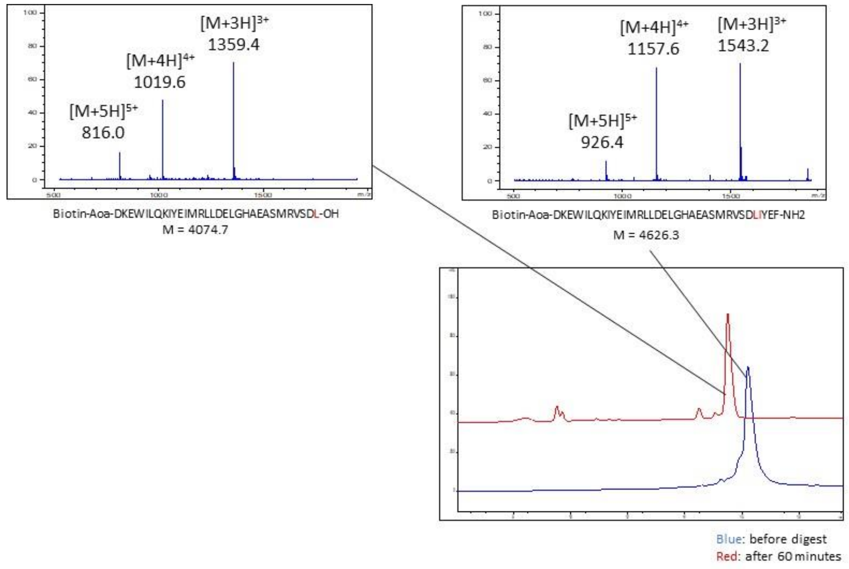

Figure S2. HPLC chromatogram and ESI-mass spectra of LW32.4 before and after pepsin digest.

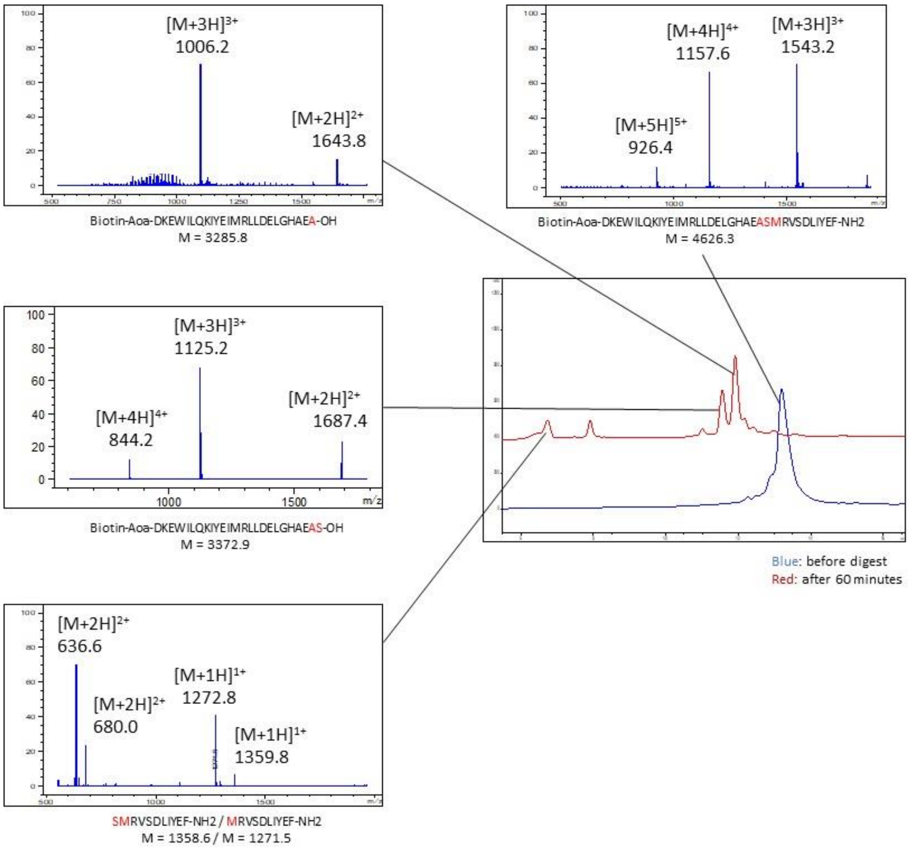

Figure S3. HPLC chromatogram and ESI-mass spectra of LW32.4 before and after neutrophil elastase digest.
